# Applications of Polymeric Membranes with Carbon Nanotubes: A Review

**DOI:** 10.3390/membranes12050454

**Published:** 2022-04-23

**Authors:** Steve F. Nitodas, Mrinaleni Das, Raj Shah

**Affiliations:** 1 Department of Materials Science and Chemical Engineering, Stony Brook University, Stony Brook, NY 11794, USA; 2 Koehler Instrument Company Inc., Bohemia, NY 11794, USA; mrinaleni.das@stonybrook.edu (M.D.); rshah@koehlerinstrument.com (R.S.)

**Keywords:** carbon nanotubes, vertically aligned carbon nanotubes, nanocomposite membranes, water purification, desalination, gas separations, energy storage, nanostructured carbon

## Abstract

Nanomaterials have been commonly employed to enhance the performance of polymeric membrane materials that are used in several industrial applications. Carbon nanotubes (CNTs) have gained notable attention over the years for use in membrane technology due to their anti-biofouling properties, salt rejection capability, exceptional electrical conductivity, and mechanical properties. This paper aims to discuss some of the recent applications of CNTs in membrane technology and their effect on a larger scale. The paper reviews successful case studies of incorporation of CNTs in membranes and their impact on water purification, desalination, gas separations, and energy storage, in an effort to provide a better understanding of their capabilities. Regarding the future trends of this technology, this review emphasizes improving the large-scale production processes and addressing environmental and health-related hazards of CNTs during production and usage.

## 1. Introduction

In recent years, innovation inspired by nature has become a huge part of our lives. The skillful manipulation of different materials at the scale of 1–100 nanometers is proven to be the solution to many long-lasting problems where membranes are employed, such as water filtration and gas separations. Nanostructured carbon materials are gaining popularity every day because of their unique atomic structure and outstanding physical and mechanical properties (e.g., thermal conductivity, tensile strength, porous structure). Nanostructured carbon includes various carbon nanofibers, carbon nanotubes (CNTs), fullerene, and graphene. Among these, carbon nanotubes are composed of cylindrical graphite sheets rolled up in a cylindrical structure. They are characterized by exceptional mechanical properties and high surface area that renders them an ideal candidate material to separate and filter out unwanted nanoparticles and contaminants [1,2].

Although the development of “artificial” membrane technology started around the 1960s, it did not gain popularity until a few decades ago when defined regulations and demonstration of cost-effective separation capability helped it to become mainstream [3,4]. Membrane technology is nowadays heavily used in the field of water filtration [5,6,7,8,9], air purification [10,11,12], and energy storage [13,14]. Over the years, researchers focused on polymer, inorganic, and hybrid mixed-material-based membranes [15,16,17]. Polymeric membranes are known for their tensile strength, high separation rates, and low production cost, and they are mostly utilized in gas and liquid separations [15,18]. Nevertheless, the lack of uniform pore distribution makes them inefficient in environments where the volume of liquid/gas to be filtered cannot be easily controlled and the targeted removal of contaminants may not be attained [19]. Consistent narrow pores achieved in membranes with carbon nanotubes address this issue and render the filtration process more controllable in terms of removal of specifically sized particles [15,20,21]. However, the production cost of CNTs and nanostructured carbon is relatively high, making them unattractive to big investors and industrial end users [21]. Therefore, this study reviewed the research studies that have been implemented to demonstrate the added technological value of CNT-based membranes for the aforementioned applications, which can act as an indirect tool toward the reduction of the production cost.

Recent studies have investigated carbon nanotubes for improving stability and decreasing the cost of production because of their interconnected pore structure, large surface area, and higher mechanical stability. In addition, the thermal stability and controllable pore diameter of carbon nanotubes facilitate their utilization in different separation methods [6,22].

Although in the beginning, the CNTs were developed as an additive to improve the mechanical strength, permeability, and resistance of polymeric membranes; however, due to their exceptional properties, researchers experimented with their potential in water purification such as desalination, wastewater treatment, and pollutant filtration [3,16,23]. Their usage in areas such as gas separations and energy storage is somewhat a new approach. The strong association of CNTs with membrane technology is due to the unique design of CNTs that can tailor the size of the membrane pores, thus allowing size-exclusion separation, as well as the hydrophobic nature of carbon nanotubes that can facilitate the fast transport of polar water molecules.

## 2. Types of CNT-Based Membranes

Currently, there are two types of nanotube membranes available in the market: (i) vertically aligned carbon nanotube (VA-CNT) membranes and (ii) mixed-matrix carbon nanotube (MM-CNT) membranes [24].

(i) *Pure-CNT membranes:* VA-CNT membranes have been investigated since 1998 when Che et al. designed a cylinder with a diameter of 20 nm by aligning carbon nanotubes vertically using the chemical vapor deposition method (CVD) [13]. A VA-CNT membrane consists of unique microstructured cylindrical pores, which force fluids to pass through hollow CNT walls or CNT bundles; their consistent isoporous structure enables them to be utilized in numerous filtration processes [25,26,27]. Buckypaper CNTs have been also investigated by researchers because of their simple fabrication process. These membranes are synthesized from CNT powders (pristine carbon nanotubes); however, their mechanical performance is not compelling due to the random dispersion of CNTs in the membranes. The use of proper polymer binder on CNT surface (functionalization) can significantly enhance their performance due to the resulting uniform dispersion of carbon nanotubes in the membrane matrix [28,29,30].

**Figure 1:**
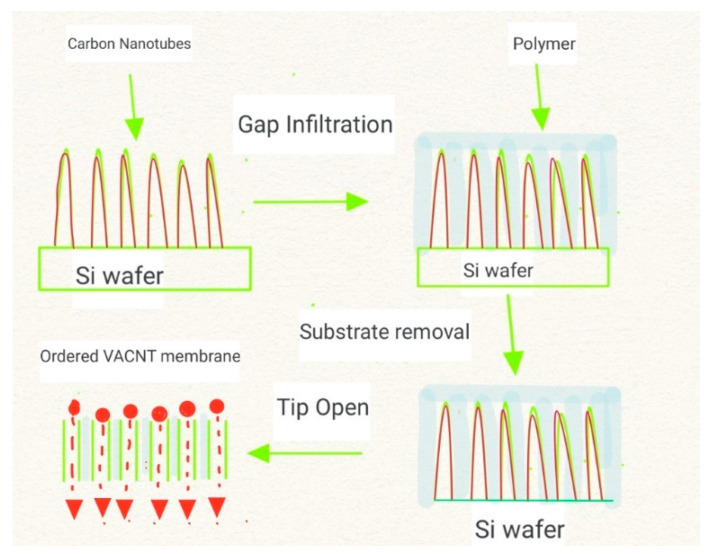
Synthesis process of VA-CNT.CVD is initially employed for the synthesis of VA-CNTs on a silicon (Si) wafer pre-coated with metal catalytic particles. Subsequently, gap infiltration takes place with polymers (e.g., epoxy or polystyrene) under vacuum. The substrate is then removed by etching, and finally, a tip opening is carried out by mechanical polishing, ion etching, or plasma etching [25].

**Figure 2:**
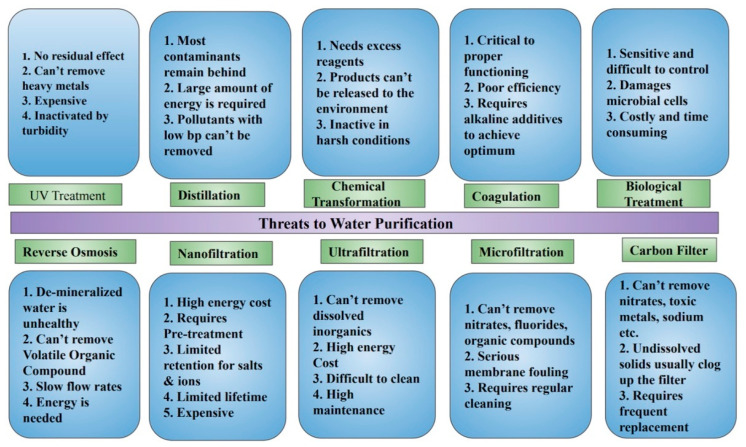
Major shortcomings of conventional purification systems (Reproduced with permission from Elsevier; adapted from Das et al., 2014 [23]).

Although CVD is the most common way to synthesize VA-CNT (Figure 1), some studies suggest using a plasma-enhanced CVD (PECVD) process to design a highly ordered vertical structure by manipulating the high electrical polarizability caused by the external electric field in the PECVD chamber. This method can produce nanotubes ranging from a diameter of 100 to 1 nm, and the controllability of the pore size makes it an excellent candidate for membrane distillation [25,31]. The frictionless wall of the highly porous structures of VA-CNT membranes leads to a larger flow of liquid due to the slippage effect; however, when pore density is less than (6 ± 3) × 10^10^ cm^−2^, the fluid transport decreases dramatically [32].

(ii) *Mixed-Matrix CNT (MM-CNT) membranes:* MM-CNT membranes are heterogeneous structures that consist of several layers of inorganic fillers in a random order in a polymeric matrix (Figure 2) [33,34]. These membranes were first designed in 2006 by blending multi-wall CNTs (MWCNTs) with poly (sulfone) (PSF) to improve the functionality of ultrafiltration membranes by Choi et al. [35,36].

The tubular structure and frictionless capacity of these membranes reduce energy consumption and induce the formation of differently sized pores which are sensitive (selective or not, depending on the application) to pollutants and salt [37,38,39]. A general comparison between the VA-CNT membranes and the MM-CNT membranes is shown in Table 1.

## 3. Detailed Applications of CNT Membranes

### 3.1. CNT Membranes in Water Purification

Clean water might be easily available to many parts of the world, but it constitutes a luxury to most parts of Africa, Asia, and some parts of Europe. According to the World Health Organization, approximately 2 billion people consume contaminated water, and by 2025, half of the world’s population will live in water-stressed areas [40]. In recent years, water pollution due to heavy metal ions, such as lead and zinc, has raised concerns in industrial areas [41,42,43]. The traditional lack of clean water supplies, the emerging concerns about micro/nano-pollutants, and climate change also jeopardize the availability of clean water for households, agriculture, and industrial sectors [23].

Figure 2 presents the current drawbacks encountered in various water purification systems that need to be overcome with more sophisticated methodologies. A study conducted by Baek et al. proposed the use of VA-CNT membranes for water purification, claiming a three times higher water flux than the traditional UF membranes and 70,000 times higher water transportation than conventional no-slip flow systems [44]. During the investigation of Baek et al., the VA-CNT membrane was synthesized using Fe as a catalyst through a water-assisted thermal CVD onto a Si wafer, which had an effective area of 0.1 cm². The proposed design had a smaller pore diameter of 4.8 ± 0.9 nm compared to “traditional” ultrafiltration (UF) membrane pores of 5.7 ± 2.5 nm (Table 2). The proposed design also exhibited a more hydrophobic surface, as can be concluded from the contact angle values in Table 2. Although the weaker tensile strength and Young’s modulus might render the VA-CNT membrane mechanically inferior to UF membranes, the latter can operate only at pressures under 2.8 MPa. Hence, the proposed design of CNT membranes can be used as a substitute for UF membranes [44].

**Figure 3:**
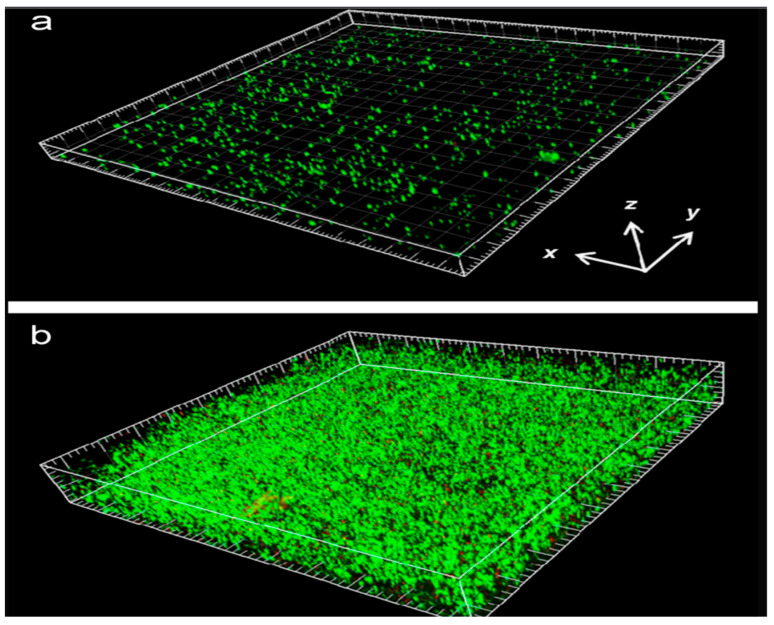
Confocal laser scanning microscopy (CLSM) images after biofouling occurrence: (**a**) VA-CNT membrane vs. (**b**) UF membrane (green: live cells) (Reproduced with permission from Elsevier; adapted from Baek et al., 2014 [44]).

One of the major testing criteria of this study was the anti-biofouling tendency of CNT-membranes. The researchers used *Pseudomonas aeruginosa* PAO1 GFP as a bacterial strain with a concentration of 1 × 10^7^ CFU/mL (CFU: colony-forming unit), and the membrane was conditioned using 10 mM NaCl, 10 mM sodium citrate, and 0.1% tryptic soy broth. The VA-CNT membrane faced a less severe decrease in permeate flux and a smaller bacteria concentration (4 × 10^5^ CFU/mL as opposed to 8 × 10^7^ CFU/mL in UF membranes), which is most likely caused by the physical damage or oxidative stress from the CNT membrane surface (Table 2) [44]. The same conclusion on the bacteria accumulation by the two membrane types can be also derived from Figure 3, which shows smaller bacteria concentration attached on the VA-CNT membrane. 

Another study conducted by Kang et al. confirmed that the size of the diameter has an effect on the antibacterial effect of the membranes; single-wall carbon nanotube (SWCNT) membranes are more toxic to the bacteria than multi-wall carbon nanotube (MWCNT) membranes [45]. Other studies also claim that the presence of reactive oxygen also affects the cytotoxicity of the CNT membranes positively [46,47], that is, it reduces their toxicity.

**Figure 4:**
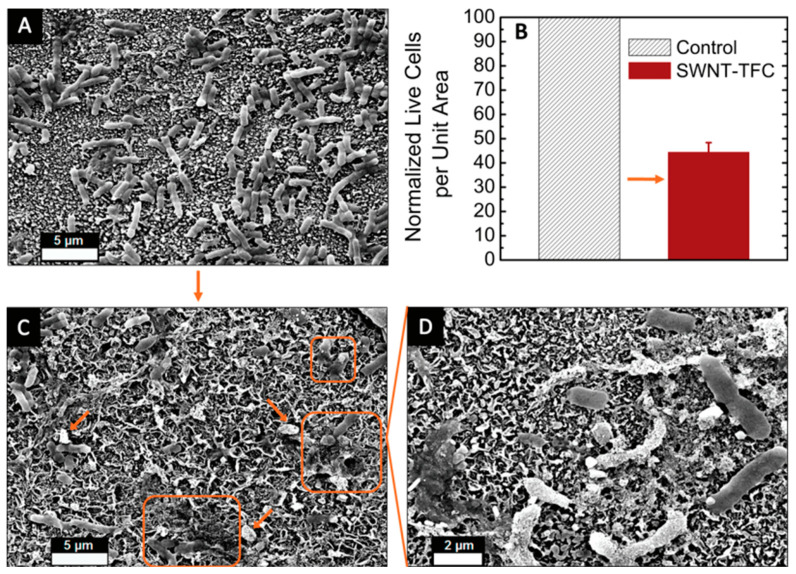
SEM micrographs displaying *E. coli* at the surface (**A**) control membrane and (**C**) an SWCNT-functionalized membrane at the end of the cytotoxicity test (some cells with lost integrity are highlighted in orange). (**D**) Magnified view of the surface of SWCNT-TFC membrane with *E. coli* cells [45]. (**B**) Colony-forming units (area units are in μm²) enumerated from *E. coli* bacteria resuspended from functionalized SWCNT-TFC membranes (red) normalized with those of control membranes (shaded). The tests involved incubation of bacterial suspension in contact with the membrane surface for 1 h in 0.9% NaCl at room temperature (23 °C). Bars with standard deviation represent the average of three separately cast and reacted membranes (Reproduced with permission from ACS Publications; adapted from Tiraferri et al., 2011 [48]).

A study conducted by Tiraferri et al. studied the antimicrobial properties of SWCNT membranes, which can be used in water purification [48]. In this study, the researchers covalently bonded the cytotoxic SWCNT to the surface of polyamide membranes to induce anti-microbial activities. The thin-film composite (TFC) polyamide membrane was developed using interfacial polymerization of polyamide onto commercial polysulfone (PSF) ultrafiltration membranes (PS20), and the CNT was treated with ozone (O_3_). Many studies have shown that ozonolysis decreased particle size and induced defects in nanotube walls that facilitate carboxylic functionalization and dispersion in water [49,50]. When the *E. coli* K12 was introduced to the CNT membranes, the ozonized CNT membrane showcased a significant reduction in bacteria’s survival rate (>95%), as can be seen in Figure 4 [48].

Figure 4B confirms that microbial concentration in SWCNT membranes was significantly lower (~44%) than the control membrane. Comparing Figure 4A,B, it can be said that most of the bacteria in the control membrane appeared to be healthier than the bacteria in the SWCNT-functionalized membrane, which further proves the cytotoxicity of SWCNT-functionalized membranes that is most likely caused by the homogeneous distribution of SWCNTs. Another major concern related to functionalization is if it leads to a decrease in the permeability coefficient; however, the study confirmed that there was no significant change in the purification rate compared to the control group [48].

**Figure 5:**
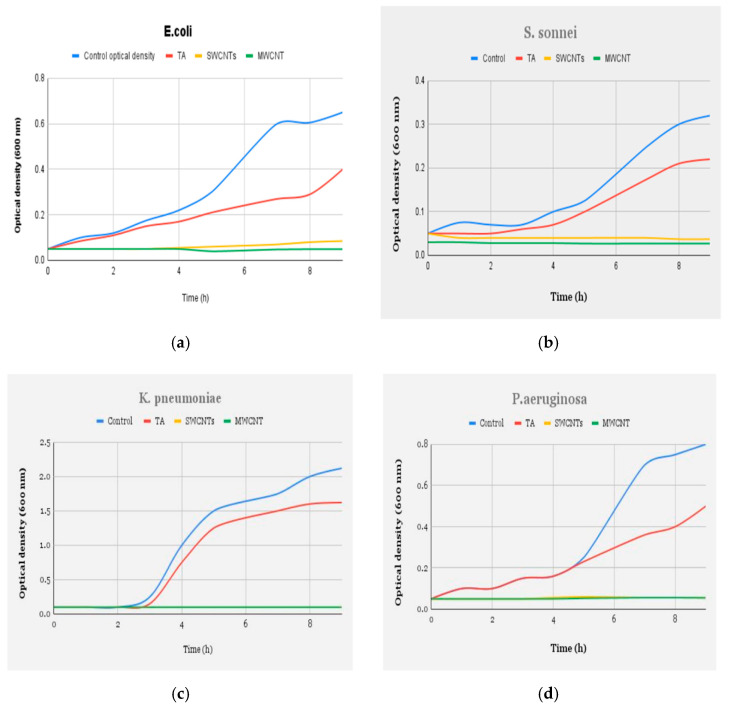
Growth curve measurements of (**a**) *E. coli*, (**b**) *S. sonnei*, (**c**) *K. pneumoniae,* and (**d**) *P. aeruginosa* in minimal broth showing antibacterial effects of SWCNTs/MWCNTs (90 μg/mL) and their amelioration on the antioxidant (TA)-coated CNTs (90 μg/mL) as compared with the control sample. The cell density of various bacterial species used in the treatments ranged between 1 and 1.6 × 10^7^ CFU/mL (Reproduced with permission from ACS Publications; adapted from Rajavel et al., 2014 [46]).

Another study conducted by Rajavel et al. focused on the effectiveness of single-wall, multi-wall, and antioxidant tannic acid (TA) functionalized CNTs for use in membranes to reduce bacterial viability [46]. The study found that all the CNT types exhibited remarkable cytotoxic activity against all the tested bacterial pathogens maintained in the minimal medium, as observed in Figure 5 (agar medium was used as a control). This work also confirmed that membranes become toxic in the presence of UV light, and there is a significant drop in cytotoxicity in the dark. The study also found a strong correlation between the presence of oxygen species and the increase in anti-biofouling activities [46]. A study conducted by Wang et al. concluded that functionalized CNTs (f-CNTs) mixed with hybrid polyethersulfone (PES) achieved an antibacterial rate close to 100% through damaging the cell of bacteria (*E. coli*) using two reduced oxygen atoms from the hybrid membrane and forming hydrogen peroxide [51]. Another study proposed the use of polyethylene glycol (PEG) with CNTs to reduce cytotoxicity without lowering the antibacterial properties of the membrane. The proposed design was able to reduce bacterial density by 91.5% within a short span of time, and the toxicity level was minimal [52].

Another application of CNTs in water purification lies in the utilization of the physicochemical properties of CNT membranes to remove toxic heavy metals, refractory contaminants, and radioactive particles from water, thus reducing environmental pollution caused by these particles. The existing membrane technologies are unable to reject dissolved organic pollutants that have lower molecular weights (<100 Da) [53]. Several research studies have shown that CNTs are the feasible, cost-effective, and efficient choice for electroactive membrane technology [54,55,56].

Most research conducted on heavy metal removal concentrates on iron, zinc, manganese, and arsenic removal. However, there are not many technologies available to address the growing concern with antimony (Sb) removal. A recent study conducted by Liu et al. utilized the TiO_2_-CNT membrane to remove Sb(III) and transform it into less toxic Sb(V). The study found that the electric field created by the CNT membranes accelerates the SB(III) conversion process, and the proposed system operated at an efficiency greater than 90%. The proposed design also addresses the exhaustion of membrane after multiple usages; however, this design can be easily regenerated and maintain efficiency using NaOH solution [57]. In this study, multi-walled CNTs were modified using TiO_2_ through a simple electrosorption-hydrothermal process. They tested the proposed design in a practical setting where the researchers spiked tap water with Sb(III) and applied voltage of 2 V, and the recorded removal efficiency was lower than the rate found during laboratory experiments. However, this process was more energy-efficient (0.01 kwh/m^3^), and consistent regeneration rendered the technology noteworthy [57].

Another recent study explored the use of CNTs in removing chromium—Cr(VI)—particles from wastewater and converting them into Cr(III), under the application of electric field, thus reducing the toxicity level [58]. The proposed design by Liu et al. utilizes nanoscale polyaniline (PANI)-functionalized CNTs that are able to increase the efficiency up to 70% at a voltage of 2.5 V, which is significantly higher than current existing technologies. The improvement is most likely due to the exceptional electrical conductivity, increased available active sites, and smaller pore size of CNTs [58].

The amine and imine moieties of PANI help to reduce Cr(VI) to Cr(III), which is a less toxic particle. This reduction in the presence of PANI was found to be effective in an acidic environment (PH 1–3), but to further remove Cr(III) efficiently from wastewater with PANI, a basic environment is required. Because of this shortcoming, the researchers were not able to attain the desired outcome of Cr(III) removal [58]. However, other studies argued that it is cost-effective to enhance the Cr(VI) conversion process to Cr (III) and subsequently utilize PANI-based composites, such as in polystyrene (PS), to remove Cr(III) since it is less harmful than Cr(VI) [59,60]. Since positively charged bulky PANI, if used on its own, repels electrostatically positive Cr(III), PANI-PS composites were found to efficiently sequester positive Cr(III) due to the negative surface created in the PANI by confinement effects in the nanopores of PS [60].

All of the aforementioned studies solidify the potential of CNT-based membranes in water purification and showcase their ability to address the issue associated with current membrane technology by reducing bacterial growth, increasing water flux, and removing heavy metal ions from water in a cost-effective and efficient manner.

### 3.2. CNT Membranes in Desalination Technology

Over the years, the urge to desalinate and remove micro/nano-pollutants in cost-effective ways has been one of the major challenges that membrane scientists faced. Traditional microfiltration (MF) and ultrafiltration (UF) membranes (e.g., polyethersulfone (PES), polysulfone (PSF), and polyvinylidene fluoride (PVDF)) cannot filter out nano-pollutants because of larger pore size, resulting in only a 20–50% natural organic matter rejection rate. Membranes such as polyamide (PA) and cellulose acetate (CA), which are used for desalination, are vulnerable to degradation in the presence of chlorine and need energy-consuming pre-treatment for them to function [61,62].

**Figure 6:**
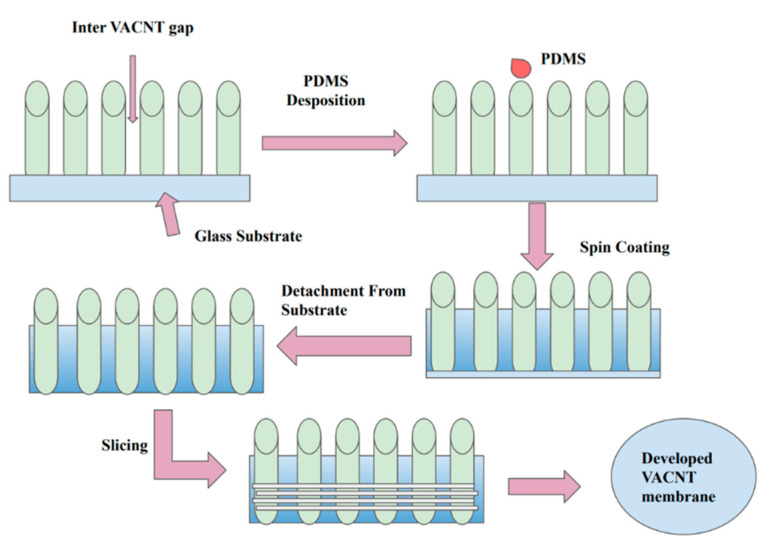
Fabrication process of VA-CNT membrane [63]: VA-CNT grown on silicon support is glued to glass substrate. PDMS is then added through spin coating and cured through heating in a vacuum oven; the volatile part of the PDMS is evaporated through spin coating. Silicon and glass substrates are removed using mechanical peeling (microtome machine).

A study conducted by Trivedi et al. investigated the potential of VA-CNT membranes in desalination technology and found that they increased the salt rejection rate to a great extent [63]. In this study, vertically aligned carbon nanotubes with a density of 5 × 10^9^, 1 × 10^10^, 5 × 10^10^, and 1 × 10^11^ cm^−2^ were employed, which were grown on a Si wafer that was subsequently coated with 50% (*w*/*w*) poly (dimethylsiloxane) (PDMS) and then sliced and developed into a VA-CNT membrane (Figure 6).

**Figure 7:**
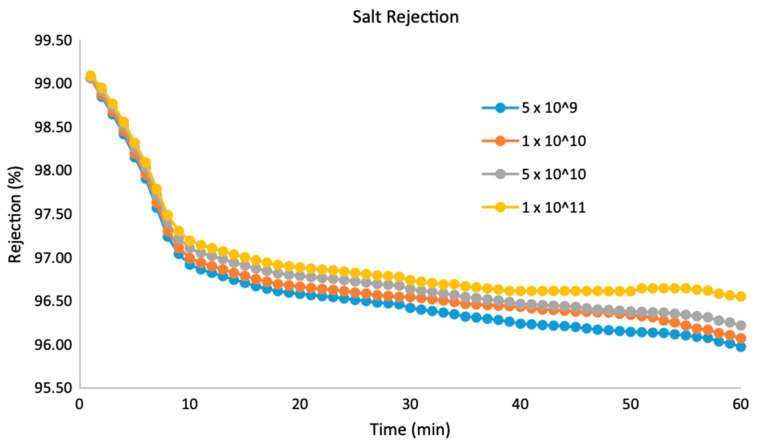
Salt rejection vs. time graph for different CNT densities of VA-CNT membranes (density is given in number of nanotubes/cm^2^) [63].

The study compared the maximum fluxes induced by these membranes and found that the membrane with the highest density has the maximum flux of 1203 LMH (liters per square meter per hour). From these data, it can be concluded that increasing density helps to attain a higher flow rate and enhanced anti-biofouling properties without damaging the salt rejection properties [63]. The antifouling properties of CNT-based PDMS were also verified by Cavas et al. [64]. According to Trivedi et al. [63], the two main reasons behind salt rejections are (i) the inner diameter of CNTs and (ii) the surface charge of the membrane. The PDMS is negatively charged; hence, the Na^+^ ions get trapped by the surface, reducing the salt concentration. This method showcased an incredible result of approximately 96% salt rejection by all of the membranes studied (Figure 7). This finding is also enhanced by the fact that the process of the CNT-based membrane fabrication in this study can be scaled up in a relatively straightforward manner as the spin coating and methods related to silicon processing have been established in the industry to a great extent for various applications [65].

Density is given in number of nanotubes/cm^2^.

Another study conducted by Thomas et al. also found similar results and determined that a carbon nanotube with a diameter of 1.1 nm is best suitable for desalination technology [66]. To achieve the most “realistic” simulation environment, the researchers used a one-way barrier (semi-permeable membrane) that blocks the passage of certain ions from the saltwater reservoir to the freshwater reservoir. This study used pressure ranging from 5 MPa to 400 MPa to investigate its effect on the CNT membranes. It was observed that as the pressure decreased, the salt rejection rate increased. The CNT membrane with a diameter of 1.09 nm rejected nearly all Cl^−^ and 90% of Na^+^, the membrane with a diameter of 1.36 nm rejected about 90% of Cl^−^ and 60% of Na^+^, and the membrane with a diameter of 1.63 nm rejected 50–80% of Cl^−^ and 20–40% of Na^+^. The Na^+^ rejection rate is lower due to its smaller ion size (radius = 98 pm), whereas the Cl^−^ is a relatively bigger ion with a radius of 181 pm. However, the study claimed that the charge separation across the membrane would equalize the permeability of each ion type, yielding the same rejection rate for both ions [64]. It can be also concluded that the decrease in the diameter of the employed CNTs leads to an increase in the salt rejection rate.

**Figure 8:**
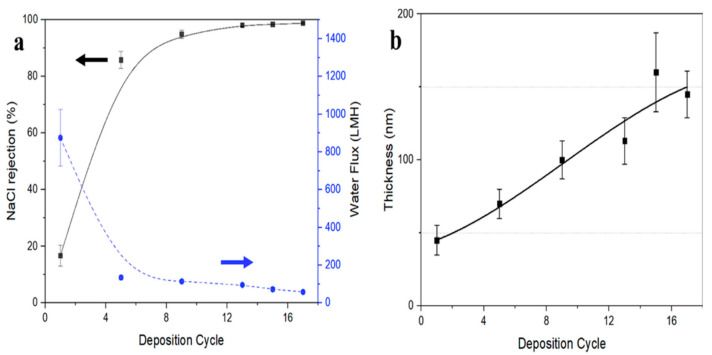
Performance of the PA/outer-wall membrane. (**a**) Water flux and NaCl rejection as a function of the number of PA formation cycles on outer-wall VA-CNT support membrane, (**b**) Thickness of PA layer on outer-wall VACNTs support membrane as a function of number of PA formation cycles (Reproduced with permission from Elsevier; adapted from Li et al., 2019 [67]).The most widely used RO membrane has a rejection rate of 98.4% and has a water permeability of 0.4–3.2 LMH∙bar^−1^, whereas the proposed VA-CNT-based membrane showcases a permeability of 4.7 ± 0.7 LMH∙bar^−1^, which is most likely caused by the weak interfacial force of water molecule and hydrophobic CNT walls [69]. The smoother outer wall of the VA-CNT-based membrane showcases a high flux rate due to reduced friction [66]. Most of the previous studies tried to improve the characteristics of the PA layer to enhance the properties of the RO membranes; however, this approach of adding CNTs as a support layer showcases an exceptional alternative for improving the RO membrane’s performance.

Another study conducted by Li et al. explored the potential of utilizing a VA-CNT membrane as a support layer for reverse osmosis (RO) membranes [67]. To densify the VA-CNT, the interfacial polymerization of m-phenylenediamine (MPD) and trimesoyl chloride (TMC) synthesized a polyamide (PA) layer on the surface of VA-CNT. The outer wall of the porous VA-CNT membrane was filled with 3 wt% MPD and 0.15 wt% of TMC. When the surface’s thickness increased by coating with larger amounts of PA, the water flux rate experienced a sharp decrease from 875.8 ± 150.3 LMH to 58.9 ± 6.2 LMH (Figure 8); however, the salt rejection rate increased from 94.9% to 98.8% [48]. Another study conducted by Corry et al. reported similar results. It stated that functionalized CNTs reduce water flux; however, the salt rejection is significantly improved because of the electrostatic repulsion. The NH_3_^+^ and COO^−^ functional groups make the pores highly charged and attract the sodium (Na) and chlorine (Cl) ions, improving the salt rejection rate by up to 95% [68].

### 3.3. CNT Membranes for Energy Storage

In recent years, there has been an uproar about switching to greener energy sources, and hence, the attempt to increase the efficiency of hydrogen-powered fuel cells has been a major topic of discussion. Among all the potential conductors, Nafion, a sulfonated fluoropolymer, has gained the attention of researchers because of its ability as a mechanically and thermally stable proton-conducting membrane in fuel cells [70].

A recent study conducted by Tortello et al. proposed the use of VA-CNT to promote the stability of Nafion membranes, when compared to those prepared with randomly oriented CNTs [70]. The incorporation of CNT is easy to obtain in the polymeric matrix without causing any disturbance to the existing properties while achieving improved mechanical and thermal stability. After many trials, the researchers concluded that VA-CNT columns of 500 μm in diameter, spaced by 500 μm of pure Nafion, with ∼100 μm height, is the optimal configuration for proton transportation. The incorporation of CNTs also increases the hydrophobicity of a surface [70,71]. Protonic and electronic transport properties were investigated in ambient and wet conditions (Table 3). When membranes are hydrated, electronic conductivity decreases while the protonic one is considerably enhanced. Protonic conductivity in ambient (dry) conditions is equal to that previously reported for randomly oriented Nafion/CNT membranes, whereas in wet conditions, it exhibited a significantly improved performance. Proton conductivity increased in the wet conditions due to the mass formation of proton-conducting pathways (water channels) along carbon nanotubes.

Another study investigated by Pilgrim et al. also showcased that VA-CNT membranes filled with epoxy are optimal for electron and proton transport and are characterized by chemical robustness, which can be utilized in artificial photosynthesis applications [72]. The proposed VA-CNT was grown on a crystalline silicon substrate in a 2.5 cm diameter single opening via CVD and had a height of 100–150 μm, an outer diameter of 15–20 nm, and an inner diameter of 5–10 nm. Epoxy was chosen as filler of the array, considering its rigidity, low cost, and antioxidant nature. This design led to twenty (20) times higher conductivity than the previous Si-Nafion-based membrane used for the specific application; this improvement is most likely caused by the reduced non-conductive space. The membrane exhibited a conductivity of 495 mS cm^−1^, and it transported a current equivalent of 5.84 × 10^−6^ A [72].

### 3.4. CNT Membranes for Gas Separations

Many water and gas purification techniques, such as membrane distillation and CO_2_ sequestration, rely on membranes. Consequently, the development of advanced membrane technologies with controlled pore architectures is essential for the attainment of an effective and cost-efficient purification. Membrane-based gas-separation technology has rapidly become a competitive alternative owing to its advantages of efficiency, low-energy consumption, and ease of operation compared with traditional gas-separation methods such as solvent absorption, cryogenic distillation, and adsorption [73,74,75]. Polymeric membranes are well known to suffer from a tradeoff between selectivity and permeability and, in some cases, exhibit low chemical resistance or are prone to fouling. Membranes based on carbon nanotubes offer a possible route to overcome these shortcomings [76,77,78]. This type of membrane has been utilized for the study of carbon dioxide capture from natural gas and flue gas, as this constitutes a critical issue due to the global warming in recent years.

A recent study on mixed-matrix (MM) membranes for CO_2_ separation was conducted by Zhang et al. and utilized multi-wall carbon nanotubes coated with N-isopropylacrylamide hydrogel (NIPAM-CNT) that were subsequently incorporated into a poly (ether-blockamide) matrix (Pebax MH 1657) [79]. The NIPAM-CNT composite additives demonstrated a uniform dispersion in the polymeric matrix. The obtained MM membranes enhanced CO_2_ permeability and selectivity in CO_2_-CH_4_ and CO_2_-N_2_ gaseous mixtures, as compared with other MM membranes utilized for this application in the past, such as pure CNT-polyimide membranes. Remarkably, MMM containing 5 wt% NIPAM-CNT increased CO_2_ permeability and selectivity by 35% and 11%, respectively, compared with the pure polymeric membrane [79]. The results of this study show that the composite additive with fast-transport nanochannels (CNT) and super water hydroscopicity (NIPAM hydrogel) is indeed an effective strategy to improve CO_2_-separation performance of MM membranes.

Khan et al. fabricated MM or MMM with MWCNTs as fillers and polymer of intrinsic microporosity (PIM) as matrix [80]. PIMs are microporous materials that are characterized by a very large volume of inter-connected pores. Since their development in 2004, PIMs have attracted a lot of attention as gas-separation membranes, sensors, or highly efficient adsorbents for organic vapors [81]. Both pristine MWCNTs and MWCNTs functionalized with poly (ethylene glycol) were utilized. Scanning electron microscopy revealed that the functionalized MWCNTs were well dispersed throughout the PIM-1 matrix compared to the one fabricated from pristine MWCNTs. With good interfacial adhesion between the polymer matrix and the functionalized MWCNT fillers, the MM membranes exhibited higher permeabilities by 80.68% for O_2_ and 53.5% for CO_2_, as compared to the pure polymer membrane, and for a filler content between 0.5 and 2 wt%. The MM membranes were also characterized by increased O_2_/N_2_ and CO_2_/N_2_ selectivities, more specifically by 28.7% and 18.8%, respectively. Above the content of 2 wt%, agglomeration of functionalized MWCNTs in the polymer matrix was observed, which hindered the fast transport of gases. Single-wall carbon nanotubes have been also investigated as filler in MM membranes for gas separations [81].

Zhang et al. showed that permeabilities of oxygen, carbon dioxide, and hydrogen through SWCNT-MM membranes showed a nonlinear trend with increasing filler loadings, which was due to the competition between impeded gas diffusion by the carbon walls and increased gas transport through the inner structure of carbon nanotubes [82]. Significant improvement in the selectivity of different gases was observed in the membranes containing SWCNTs that were purified from metal catalytic particles and functionalized with carboxyl groups (COOH) through acid treatment. A polyimide membrane with 2 wt% COOH-SWCNTs exhibited both high permeabilities and selectivities, compared to the pure polyimide membrane, locating the performance above the 1991 Robeson’s upper bound for CO_2_/CH_4_, O_2_/N_2_, and H_2_/CH_4_ [83]. The remarkably improved performance was attributed to the high purity of the modified carbon nanotubes, the CNTs’ open end caps that were facilitated by the acid treatment and allowed for gas molecules to pass through, and the CNT functionalized surface that improved the solubility selectivity.

## 4. Challenges and Future of CNT-Based Membranes

Until now, one of the major challenges in this field remains the commercialization of CNT-based membranes and the potential environmental impact. Over the years, researchers have been trying to find potential applications of CNTs; however, there has not been much research on the environmental impact of CNTs produced on a large scale. The toxic nature of carbon nanotubes has been reported in some cases; a low 0.38 μg cm^−2^ dose of single-wall CNTs can impair phagocytosis, while 3.06 μg cm^−2^ of multi-wall CNT can cause serious injury [84]. The CNTs are also easily contaminated by disordered carbon and aromatic hydrocarbons, which can be hazardous in big-scale production processes [85]. Pristine CNTs can be carcinogenic, capable of inducing lung tumors as has been reported [86]. In addition, the production of uniform and homogeneous structures of CNTs is difficult to achieve on a large scale, and this can pose a threat to commercialization [87]. As a consequence, the replacement of existing desalination technology by VA-CNT will be only possible if consistent production of CNTs is feasible, mainly with a small diameter [88,89]. These challenges can be mostly overcome if a biocompatible CNT is designed. As produced CNTs are insoluble in water, biocompatibility can be achieved by modifying the surface of CNTs with different water-soluble functional groups, such as carboxyl and hydroxyl [90,91]. Biocompatibility can open the way for the realization of medical applications of CNT-based polymeric membranes, such as artificial photosynthesis, implants, and bone tissue regeneration [72,92,93].

Most of the production process of VA-CNT membranes involves the CVD process, which is economically viable; however, it limits the use of substrates and templates for VA-CNT fabrication. A recent study proposed the use of fluidized bed CVD as a solution and claimed that this process increases the production rate and purity of the CNTs while improving the efficiency of the process [94,95]. The latter is currently used to fabricate powdered or arrays of CNTs to enable the production of VA-CNT [96].

Kim et al. investigated another way for the large-scale production process; CNT-based membranes are less flexible than traditional polymeric membranes because of their frame and plate module configuration [67]. This study suggested filling the space between nanotubes with flexible materials, such as styrene monomer blended with polystyrene–polybutadiene copolymer. This process will make the VA-CNTs more flexible, and they can compete with existing polymeric membranes [96,97,98].

## 5. Conclusions

Recent research has proven that CNT-based membrane technologies have a promising future due to the exceptional properties imparted by nanostructured carbon. However, complex designs and the feasibility of consistent large-scale production processes remain the biggest obstacles. Recent studies have focused on addressing these issues and achieving the ultimate desired outcomes. If the findings are correctly utilized, then processes based on CNT-based membranes will become more efficient, cost-effective, and environmentally friendly. Many aspects of the capabilities of CNTs remained unexplored; therefore, there is a need for researchers across the globe to further investigate methods and applications of the emerging CNT-based membranes. It is certain that in the near future, CNT-composite membranes will find their role in water desalination/purification technology and gas separations, allowing for greater flexibility and a broader perspective in addressing critical water issues. New commercial applications will be also enabled, such as in the energy field and biomedicine.

## Figures and Tables

**Table 1:** Difference between VA-CNT and MM-CNT [38].

Pure CNT (VA-CNT)	Mixed-Matrix CNT Membrane
Vertical CNT arrangement	Mixed CNT arrangement
Compact CNT network	CNT network loosely fit
The water flux rate is high	Water flux rate is moderately high
Complicated fabrication	Simple fabrication

**Table 2:** Comparison between VA-CNT-based membrane and UF membrane (Reproduced with permission from Elsevier; adapted from Baek et al., 2014 [44]).

	VA-CNT Membrane	UF Membrane
Material	CNT + epoxy	Polysulfone
Avg. pore diameter	4.8 ± 0.9	5.7 ± 2.5
Pore density (#/cm^2^)	6.8 × 10¹⁰	8.8 × 10¹⁰
Thickness (μm)	∼200	∼0.1

**Table 3:** Proton and electron conductivities obtained under different conditions for Nafion/CNT nanocomposites vs. vertically aligned-CNT/Nafion membranes (Reproduced with permission from Elsevier; adapted from Tortello et al., 2012 [70]).

	Electron Conductivity by EIS (mS/cm)	Electron Conductivity by EIS (Wet Condition)	Proton Conductivity by EIS (mS/cm)	Proton Conductivity by EIS (Wet Condition)
Nafion/CNTS nanocomposite, CNTs 5 wt%	0.364	0.197	2	5.1
VACNTs/Nafion, CNT 7 wt%	0.568	0.315	2	8.9

## Data Availability

Not applicable.
